# Photosynthetic characteristics of *Paris polyphylla* var. *chinensis* in response to different light intensities and soil water contents

**DOI:** 10.3389/fpls.2024.1521714

**Published:** 2025-01-23

**Authors:** Yaling Zhang, Rong Xu, Zhaozhao Wang, Juan Zhang, Xinghao Tang, Yu Chen, Xiao-Li Yan, Lu-Ping Qu

**Affiliations:** ^1^ College of Forestry, Fujian Agriculture and Forestry University, Fuzhou, China; ^2^ Fujian Academy of Forestry Science, Fuzhou, China; ^3^ CAS Key Laboratory of Tropical Forest Ecology, Xishuangbanna Tropical Botanical Garden, Chinese Academy of Sciences, Kunming, China; ^4^ University of Chinese Academy of Sciences, Beijing, China; ^5^ College of JunCao Science and Ecology, Fujian Agriculture and Forestry University, Fuzhou, China

**Keywords:** *Paris polyphylla*, shading, field water capacity, photosynthesis, shade-tolerant, cultivation

## Abstract

**Introduction:**

*Paris polyphylla* var. *chinensis* (Franch.) Hara (*P. polyphylla*) is a perennial medicinal plant with a reputation for therapeutic properties. It is imperative to study the photochemical processes of *P. polyphylla* in order to determine the optimal levels of shading and moisture management for its cultivation in artificial environments.

**Methods:**

In this study, six shading levels (no shading, 30%, 50%, 70%, 80% and 90% shading) and three soil water contents (20%, 40% and 60% of the soil water saturation capacity) were established to determine the appropriate shade intensity and soil moisture content for the growth of *P. polyphylla*.

**Results:**

The results showed that only the low shade groups (no shade and 30% shade) showed irreversible damage to the daily photosynthetic dynamics of the plant over the course of a day. It is important to note that excessive light can damage not only the quantum yield for electron transport (φDo) and PSII light quantum yield (Fv/Fm), but also various physiological mechanisms that can lead to overall plant damage and a decline in organic matter. A comparison of Fv/Fm during the midday period showed that the optimum shade intensity is between 50% and 70%. Low shading can significantly increase light use efficiency (LUE), but also reduces net photosynthetic rate (*P*n) and transpiration (*T*r), indicating the negative effect on *P. polyphylla* growth. Considering the balance between growth rate and damage incidence, 50% shade should be the optimal treatment for *P. polyphylla*, followed by 30% and 70% shade. It was also observed that treatment with low soil water content (20%) significantly reduced *P*n and LUE, while increasing stomatal conductance (gs) and water use efficiency (WUE). This is associated with a decrease in the light response curve, indicating that low soil moisture inhibits the growth of *P. polyphylla* and increases the likelihood of irreversible light damage, so the optimum soil moisture content for *P. polyphylla* should be above 20%.

**Discussion:**

Considering the economic benefits and the growth and health of *P. polyphylla* in artificial cultivation, it is recommended that shade be controlled at around 50% while maintaining soil moisture between 40% and 60% of water content.

## 1 Introduction

*Paris polyphylla* var. *chinensis* (Franch.) Hara (*P. polyphylla*), known as manyleaf paris rhizome and other names, is a perennial medicinal plant recognized for its therapeutic properties (Tang et al., 2022). It is included in the Pharmacopoeia of the People’s Republic of China alongside *P. polyphylla* var. *yunnanensis* (Li et al., 2023a). It has shown substantial therapeutic effects against various cancers and is primarily used for its anti-tumor, antibacterial, and anti-inflammatory properties (Thapa et al., 2022; Guan et al., 2024). Historically, the supply of *P. polyphylla* products has been highly dependent on the gathering of wild specimens. But because of habitat fragmentation and overexploitation (Cunningham et al., 2018), the resources are on the verge of exhaustion. Consequently, artificial cultivation is essential to protect wild stocks and meet market needs (Zhang et al., 2013; Tong et al., 2017). Nonetheless, challenges like slow growth rates, prolonged growth cycles, high seedling mortality (Zheng et al., 2023), and low economic returns (Zhang et al., 2004) abound. Additionally, successful cultivation requires meticulous field management, including shade, moisture, temperature regulation, and fertilization (Singh et al., 2022). Current research on its artificial cultivation is scant, mostly focusing on chemical composition, pharmacological effects and seedling breeding according to the indexing results of journal papers, but there is a lack of research on ecological aspects. Wild *P. polyphylla* prefers shade and has poor tolerance to drought and waterlogging (Wang et al., 2011). It favors cool and moist habitats (Rawat et al., 2023) such as valleys, bushes, broadleaved forests, streams, and rocks (Puwein and Thomas, 2020), thriving in well-drained humus or sandy soils (Thakur et al., 2023).

Light intensity is a crucial factor. Key physiological and biochemical processes, such as photosynthesis, transpiration, and stomatal conductance, are highly sensitive to it (Alon et al., 2022). Exceeding the saturation point can lead to photoinhibition (Shi et al., 2022; Didaran et al., 2024). Sudden light changes can trigger physiological responses (Hussain et al., 2021), and intense light affects photosynthetic enzyme activity (Li et al., 2023b) and PSII (Chotewutmontri and Barkan, 2020). Plants at different growth stages have varying light intensity requirements and tolerances, with seedlings being more sensitive (Matuszkiewicz et al., 2024). Shade nets are helpful (Wang et al., 2021), but determining the optimal shade level is challenging (Ahmed et al., 2020). It is vital to balance the potential and reversibility of photoinhibition with plant growth rates (Didaran et al., 2024). While strong shading may protect from midday light intensity but harm other times’ photosynthesis (Ali et al., 2020).

Soil moisture is vital, it affects seed germination and plant form. It’s key in photosynthesis regulation (Amitrano et al., 2022a; Dai et al., 2022; Todorova et al., 2022). Prolonged drought stress impacts stomatal conductance, transpiration rate, net photosynthesis rate, and water use efficiency (WUE) (Niu et al., 2023; Hussain et al., 2024a; Poudyal et al., 2024), leading to water potential and stomatal conductance changes (Hang et al., 2021). In addition, when plants encounter water stress, they will also take measures such as stomatal closure to reduce water loss (Kumar et al., 2013). The anatomical structure of their leaves is manifested as an increase in spongy tissue, a decrease in stomatal density and a reduction in leaf area (Amitrano et al., 2022a). High light intensity will also cause a similar response in stomatal density (Amitrano et al., 2022b). If light and water act synergistically for a long time, it will affect the distribution of stomata and leaf veins in plant leaves, thus influencing the process of photosynthesis (Harrison et al., 2020) and changing their adaptability to the environment (Amitrano et al., 2022b). Water scarcity in subtropical regions reduces plant heat tolerance (Hong and Yabe, 2017), while sufficient water can enhance stability and heat resistance, as seen in *Phoebe bournei* (Yu et al., 2023). Water deficits with heat stress inhibit stomatal opening, reduce conductance and impair functions of photosynthetic organs (Haworth et al., 2018; Qu et al., 2024a), also reducing transpiration and further inhibiting photosynthesis (Qu et al., 2020). However, improved water conditions can mitigate these adverse effects, aiding recovery and restoring physiological activity.

In this study, we experimentally imposed six levels of shading and three levels of soil water content on potted *P. polyphylla*. Our objective was to determine the optimal shading and soil water content for the artificial cultivation of *P. polyphylla*. It is hypothesized that: (1) *P. polyphylla*, a shade-loving herbaceous plant, is vulnerable to damage to its photosynthetic system when subjected to high light intensities. Low light intensity hinders plant growth by reducing photosynthesis. This study aims to identify the optimal shade level for *P. polyphylla* by evaluating the benefits and drawbacks of shading. (2) The soil water content may also influence the photosynthetic system of *P. polyphylla*, with insufficient water supply potentially restricting the plant’s growth. This study will quantify the minimum soil moisture level necessary for the artificial management of *P. polyphylla*. (3) Ultimately, this study will establish a scientific basis for managing light and soil moisture during the artificial cultivation of *P. polyphylla*.

## 2 Materials and methods

### 2.1 Plant materials and experimental settings

This study was conducted at the Fujian Forestry Research Institute (26°08′53″N, 119°17′03″E) in Fujian Province, China, which has a subtropical maritime monsoon climate. It is characterized by abundant sunshine, substantial rainfall, long summers, short winters, and a frost-free period of 326 days. The experimental plant materials were sourced from the Jian Ou Ronghui Herbal Medicine Professional Cooperative. Six-year-old healthy *Paris polyphylla* seedlings that hadn’t developed two leaf whorls were selected. During the pre-shading treatment preparation, they were transplanted into pots (with dimensions: top diameter 19 cm, bottom diameter 15.5 cm, depth 20 cm) with light substrate soil (Main components: organic peat, vermiculite, perlite and slag. Among them, the total nutrient content is ≥2%, and the organic matter content is ≥46%.) for initial growth support. Fifty seedlings were planted in the experimental site’s nursery, following conventional field management. The site had no shading sources to ensure shading experiment accuracy. After adaptation, shading intensity and soil water content tests were carried out from late April to early June, strictly following the plan. In April, the average high temperature was 23°C, and the average low temperature was 16°C. In May, the average high temperature was 28°C, and the average low temperature was 21°C. The temperature in early June is similar to that in May. During this period, the climate in this area was warm and humid, which was in harmony with the ecological characteristics of *P. polyphylla*.

The shading levels in this study were designed based on wild *P. polyphylla’s* microclimate and cultivation experience (Ramadani et al., 2024). During the shading phase, five shade gradients and a control group were established: 0% (no shading, Amb), 30% (SH30), 50% (SH50), 70% (SH70), 80% (SH80), and 90% (SH90), categorized as low (0%-30%), medium (50%-70%), and high (80%-90%) shading. To ensure consistent shading, considering the solar angle changes and seedling height, the shade net was positioned at a height of 1 m, with a horizontal coverage area of 1.5 m×1.5 m and a 50 cm vertical shade perimeter. Each shade group has replicated three seedlings, totaling 18 plants.

Subsequent to the shading experiment, a shade shelter with 80% coverage was built under the rooftop glass roof for soil moisture treatment tests, with moisture as the only variable. Drawing on local cultivation experience, soil moisture gradients were set at 20% (FC20, low humidity), 40% (FC40, medium humidity), and 60% (FC60, high humidity) of the soil’s saturated water holding capacity. Each shade group has replicated six seedlings, totaling 18 plants. Each experimental group underwent controlled water treatments and was cross-referenced against each other. The weighing method managed water supply, calculating target weight from substrate and pot mass. Pots were weighed regularly and watered every three days to maintain the set moisture level.

### 2.2 Leaf-level measurements

#### 2.2.1 Leaf gas exchange

Photosynthetic measurements were conducted using a portable photosynthesis system (LI-6800, LI-COR Biosciences, Lincoln, NE, USA) to assess the photosynthetic characteristics of *P. polyphylla* under varying light and water conditions. Measurements were taken on sunny days, based on daily dynamic measurement methods and light and temperature changes in Fuzhou, during both shaded conditions and at the end of water control treatments. The diurnal dynamics of photosynthetic physiological indices in *P. polyphylla* were recorded every two hours, from pre-sunrise (6:30 a.m.) to post-sunset (6:30 p.m.). Three mature leaves were randomly selected from each plant to measure net photosynthetic rate (*P*n), photosynthetically active radiation (PAR), transpiration rate (*T*r), stomatal conductance (*g*s), water use efficiency (WUE), and light energy use efficiency (LUE), among other indices. Light energy use efficiency (LUE) was calculated as LUE = *P*n/PAR, and leaf water use efficiency (WUE) was calculated as WUE = *P*n/*T*r. During the photosynthetic measurements, the photosynthetic photon flux was set to follow the ambient light intensity during the light control experiment, while maintaining a constant level of 900 μmol·m^-2^·s^-1^ to ensure a stable light intensity during the water control experiment. This value represents the average light intensity during the experiment and exceeds the light-saturated photosynthesis rates for *P. polyphylla*. The ambient CO_2_ concentration was both maintained at 400 μmol·mol^-1^, and airflow in the leaf chamber was auto-set without temperature or water controls. We ensured that the leaf area fully occupied the 6 cm² leaf chamber.

#### 2.2.2 Photosynthetic-light response curves

The light response curve of *P. polyphylla* was measured with three replicates leaves of three different seedlings for each treatment. Mature, fully expanded leaves of *P. polyphylla* were randomly selected, and the LI-6800 was used to fit the photosynthetic response curve for leaves in the soil moisture treatment group. Light intensities ranged from 1800 to 0 μmol·m^-2^·s^-1^ in decreasing order: 1800, 1500, 1200, 1000, 800, 600, 400, 200, 100, 50, and 0 μmol·m^-2^·s^-1^. The right-angle hyperbolic correction model for plant photosynthesis response to light is expressed as follows (Ruan et al., 2022):


(1) $$P_{\text{n}}=\alpha \frac{1-\text{β}I}{1+\text{γ}I}I-{\text{R}}_{\text{d}}$$

where α is the initial slope of the photoresponse curve (dimensionless), β and γ are coefficients (in m^2^·s·μmol^–1^), *I* is photosynthetically active radiation, and R_d_ is dark respiration. If the coefficient β = 0 and let γ = α/*P*_nmax_, then equation (1) will degenerate into a right-angle hyperbolic model.

The first order derivative of Eq:


(2) $$P_{\text{n}}=\alpha \frac{1-2\text{β}I-\text{βγ}I^{2}}{{\left(1+\text{γ}I\right)}^{2}}I-{\text{R}}_{\text{d}}$$

Since the first-order derivative of (1) can be equal to 0 and its second-order derivative can be less than 0, there must be a pole in (1), i.e., there must be a saturated light intensity and a maximum net photosynthetic rate. Therefore, when (2) equals 0, if the saturated light intensity of the plant is expressed by *I*_sat_, then there is:


(3) $$I_{\text{sat}}=\frac{\sqrt{(\text{β}+\text{γ})/\text{β}}-1}{\text{γ}}$$

If the maximum net photosynthetic rate corresponding to the saturated light intensity of the plant is expressed as *P*_nmax_, then we have:


(4) $$\;P_{\text{nmax}}=\alpha {\left(\frac{\sqrt{\left(\text{β}+\text{γ}\right)}-\sqrt{\text{β}}}{\text{γ}}\right)}^{2}-{\text{R}}_{\text{d}}$$

The light compensation point of the plant is denoted by *I*_c_, and we have.


(5) $$I_{\text{c}}=\frac{\text{α}-{\text{R}}_{\text{d}}-\sqrt{{\text{R}}_{\text{d}}^{2}{\text{γ}}^{2}+{\text{α}}^{2}-2{\text{αγR}}_{\text{d}}-4{\text{αβR}}_{\text{d}}}}{2\text{αβ}}\;$$

#### 2.2.3 Measurements of chlorophyll fluorescence

Chlorophyll fluorescence measurements were performed using a Pocket-PEA portable plant efficiency analyzer (Hansatech, UK). The measurement time and frequency were consistent with the photosynthetic measurements. After 30 minutes of dark adaptation, chlorophyll fluorescence parameters such as initial fluorescence (Fo), maximum fluorescence yield (F_m_), maximum photochemical efficiency (Fv/F_m_), and actual photochemical efficiency (PSII), along with OJIP curves, were measured. The OJIP curves were induced by a 3000 μmol·m^-2^·s^-1^ saturating red light of 1-second duration, with an initial measurement frequency of 108 flashes per second. The O, L, K, J, I point on the OJIP curve correspond to 10 μs, 100~200 μs, 300 μs, 2 ms, and 30 ms, respectively, and the P point represents the maximum fluorescence moment. The OJIP curves were standardized using the following formulas:


(6) $$\text{VO}-\text{P}=\left(\text{Ft}-\text{Fo}\right)/\left(\text{Fm}-\text{Fo}\right)$$

(7) $$\text{VO}-\text{J}=\left(\text{Ft}-\text{Fo}\right)/\left(\text{FJ}-\text{Fo}\right)$$

Where VO-P represents the standardized relative variable fluorescence intensity at the O-P point, Ft is the fluorescence value at any given moment, Fo is the fluorescence value at the O point, Fm is the fluorescence value at the P point, and VO-J represents the standardized relative variable fluorescence intensity at the O-J point, with FJ being the fluorescence value at the J point. Quantum yield for electron transport: φ*E*o = (1−V_J_) × (Fo/Fm); Quantum yield for thermal dissipation: φ*D*o = Fo/Fm, where Fo and Fm represent the fluorescence values for times 20 μs, and 300 ms, respectively.

### 2.3 Data analysis and statistics

One-way analysis of variance (ANOVA) was used to examine the significant difference response of *P. polyphylla* photosynthetic characteristics to different shading or water level. A repeated ANOVA was employed to ascertain the existence of a significant difference between the shading treatments with daily variation. We measured the irreversible damage suffered by the plants through the difference in Fv/Fm values between sunset time (18:30) and sunrise time (6:30). Meanwhile, the maximum damage suffered by the plants was measured by the difference in Fv/Fm values between noon time (12:30) and sunrise time (6:30). All statistical analyses were conducted using SPSS 22.0 for Windows (SPSS Inc., Chicago, Illinois, USA).

## 3 Results

### 3.1 The effect of different shading levels on the photosynthetic capacity of *P. polyphylla*

**Figure 1:**
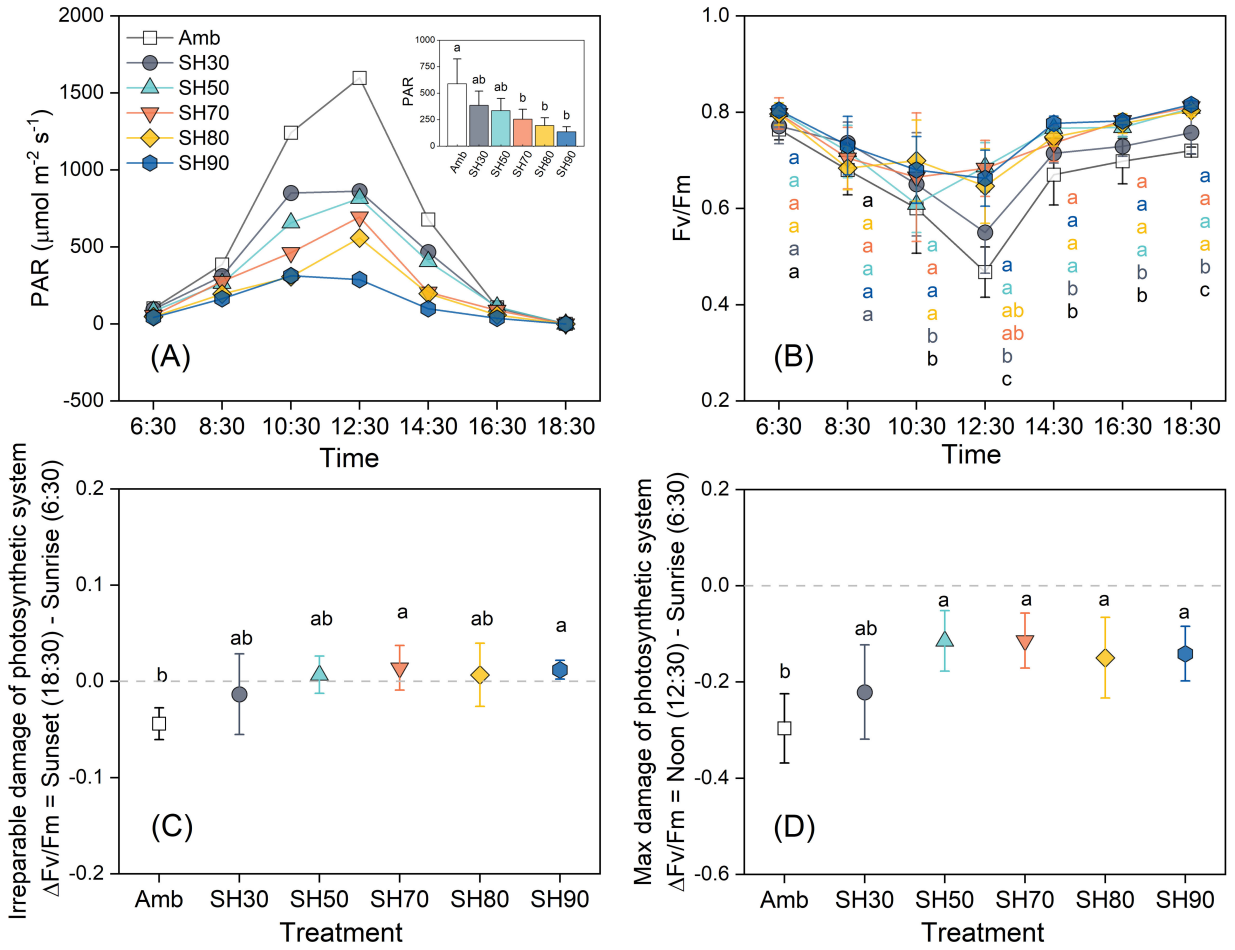
Daily variation of *Paris polyphylla* photosynthetically active radiation [PAR, **(A)**], quantum yield of PSII [Fv/Fm, **(B)**], the irreparable damage **(C)** and max damage of Fv/Fm **(D)** in different shading treatments. Values in figure b-d are means ± SE (n=3). Amb indicated the ambient, SH30 to SH90 indicated the shading 30% to shading 90%, respectively. The irreversible damage suffered by the plants was measured by the difference in the values of the maximum photochemical quantum yield of Photosystem II (Fv/Fm) between the sunset time (18:30) and the sunrise time (6:30). Meanwhile, the maximum damage suffered by the plants was measured by the difference in the Fv/Fm values between the noon time (12:30) and the sunrise time (6:30). Different letters indicate significant differences (*P*<0.05) in one-way ANOVA (Duncan test), and the small figure in **(A)** was statistic by repeated ANOVA.

Light intensity exhibits a distinct daily dynamic pattern. It progressively ascends until reaching its zenith at midday (12:30) with a value of 1597 μmol·m^-2^·s^-1^, and subsequently descends to its nadir (1 μmol·m^-2^·s^-1^) in the evening (**Figure 1A**). Notably, the daily variation of Fv/Fm exhibits an inverse relationship with light intensity. The most substantial increment in light intensity transpires precisely at noon, concomitantly, Fv/Fm experiences the most prominent decrease. The troughs are manifested in the 50% and 70% shade groups at 10:30. Nevertheless, as depicted in **Figure 1C**, certain light-induced damages prove to be reversible, thereby intimating that shading does not exert a substantial adverse impact on plant health. In the remaining groups, the trough emerges at 12:30, signifying that light-inflicted damage augments in tandem with light intensity. As light intensity wanes, Fv/Fm gradually rebounds and attains a stable state (**Figure 1B**). During the period from sunrise to midday, ΔFv/Fm remains in the negative domain across all shade levels, with significant disparities only observable between the no-shade and over 30% shade groups (**Figure 1D**). At sunset, ΔFv/Fm assumes a negative value solely in the no-shade and 30% shade groups, implying the occurrence of irreversible damage, whereas the other groups exhibit positive values, suggesting the presence of reversible damage. The most substantial daily augmentation in Fv/Fm is manifested in the 50% and 70% shade groups, thus indicating that the optimal light intensity for *P. polyphylla* resides within this range (**Figure 1C**).

### 3.2 OJIP curves and photosynthetic characteristic of *P. polyphylla* response to different shading levels

**Figure 2:**
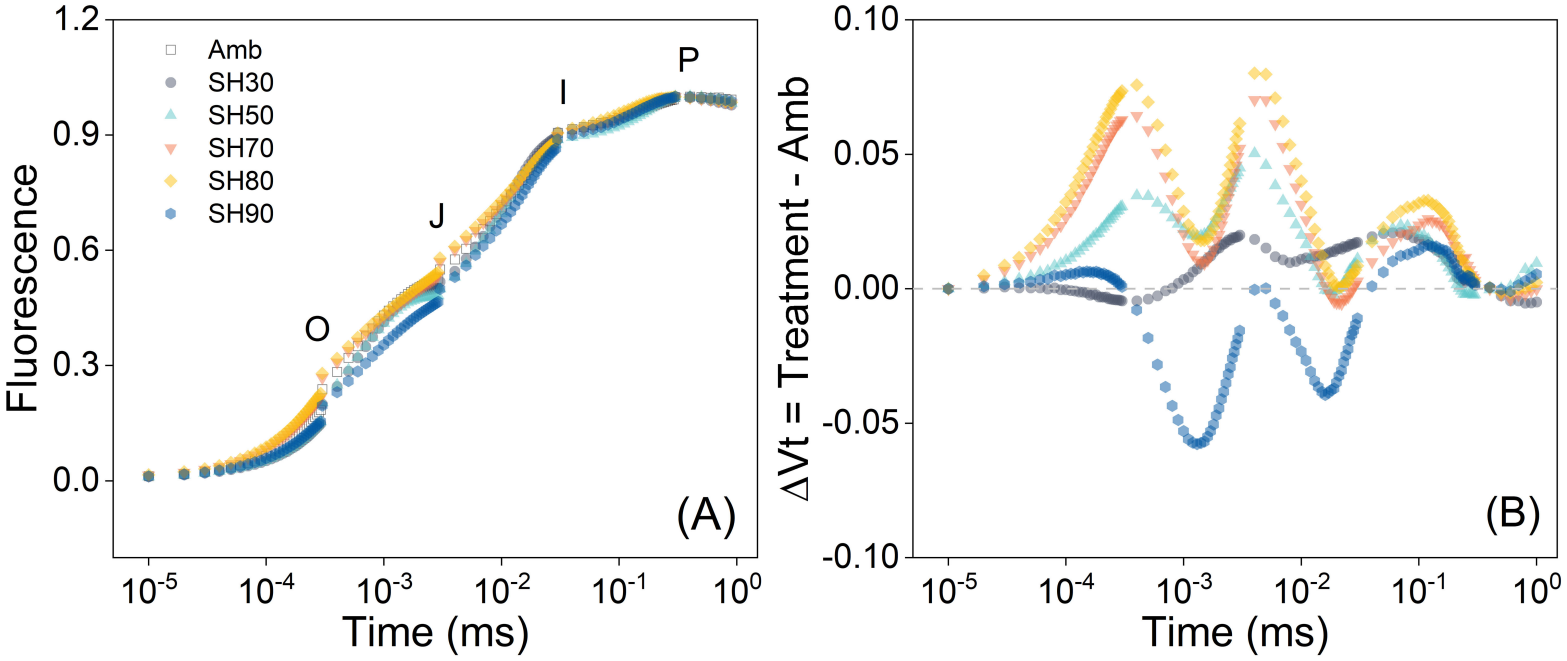
Chlorophyll a fluorescence induction curve of *Paris polyphylla* in different during development of photosynthetic apparatus **(A)** and the effect of development of PSII on the relative variable fluorescence **(B)** in different shading treatments at 12:00 a.m. Vt = (Ft – Fo)/(Fm – Fo), ΔVt was calculated by comparing of different shading treatment and ambient treatment. Values are means. Amb indicated the ambient, SH30 to SH90 indicated the shading 30% to shading 90%, respectively.

The shading treatment exerts a significant influence on the OJIP curves of *P. polyphylla* (**Figure 2**). Specifically, the 70% and 80% shade treatments yielded the largest fluorescence values, the highest electron transfer efficiency, and the strongest photosynthetic capacity. It was observed that the relative variable fluorescence magnitudes of each treatment group surpassed those of the no-shade group, with the 80% shade treatment demonstrating the highest value. As the degree of shade escalated and the light intensity diminished, the relative variable fluorescence exhibited a tendency of initially increasing and subsequently decreasing. These results imply that within a specific range of shade, there exists a positive correlation between the rate of electron transfer of the plant and the degree of shade. Among the various shade levels, 70% and 80% had the most favorable and comparable impacts on the fluorescence ability of plant leaves. Under these conditions, the leaves of *P. polyphylla* manifested the greatest light-capturing and processing capabilities, followed by the 50% shade intensity. However, when the shade intensity exceeded 80%, the plant endured excessive shading, thereby resulting in a deterioration of its maximum light-capturing and processing abilities (**Figure 2B**).

**Figure 3:**
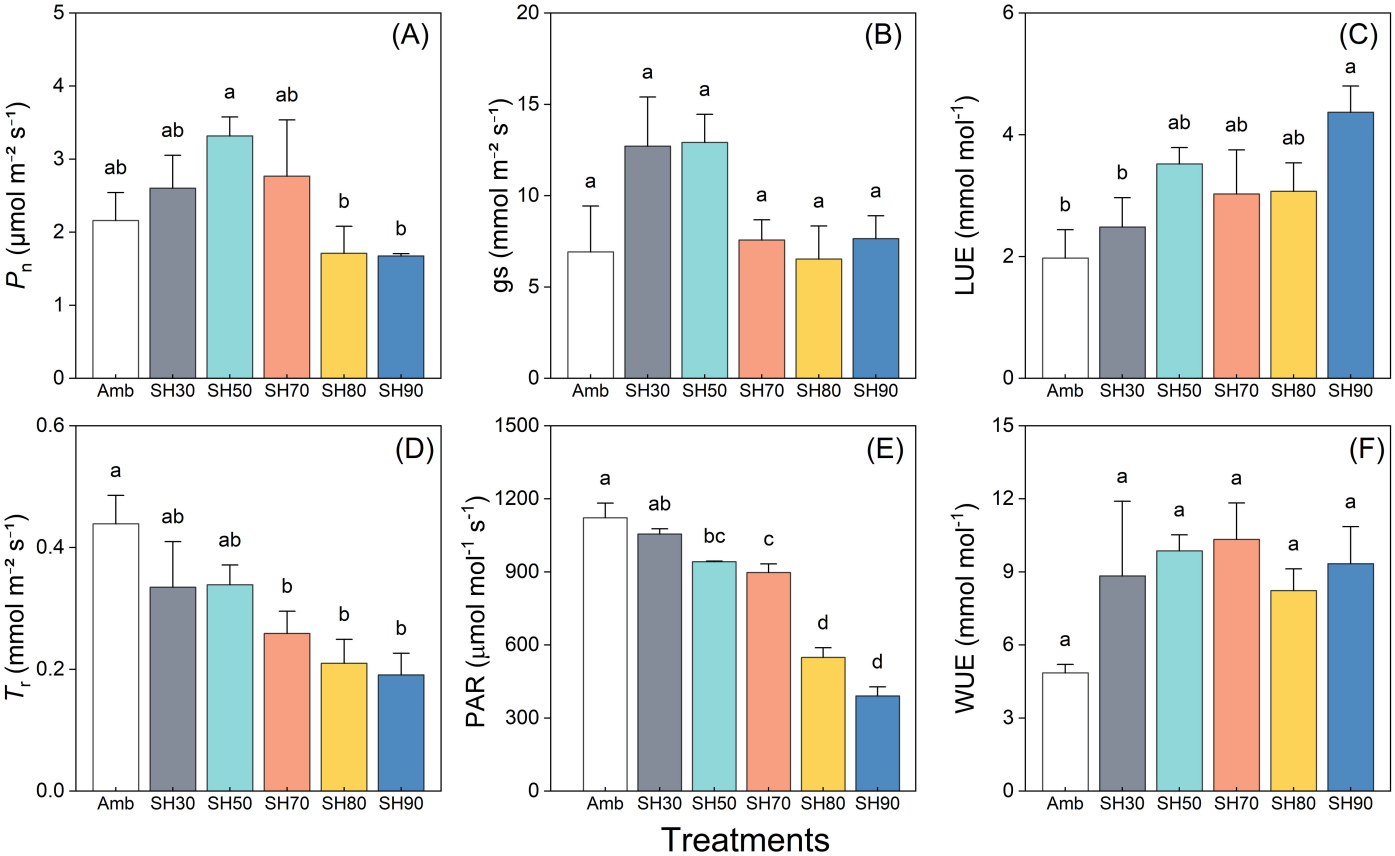
Shading effects on *Paris polyphylla* net photosynthetic rate (*P*n, **A**), stomatal conducatance (gs, **B**), light use efficiency (LUE, **C**), transpiration rate (*Tr*, **D**), photosynthetically active radiation (PAR, **E**) and water use efficiency (WUE, **F**) in 10:00 a.m. Values are means ± SE (n=3). Horizontal coordinate indicate different shading treatments, Amb indicated the ambient, SH30 to SH90 indicate the shading 30% to shading 90%, respectively. Different letters indicate significant differences (*P*<0.05) in one-way ANOVA (Duncan test).

The net photosynthetic rate (*P*n) of *P. polyphylla* was found to be substantially influenced by different shade treatments spanning a range of shade intensities from 0 to 90%. A prominent trend manifested itself, wherein *P*n initially augmented with the escalation of shade intensity, only to subsequently decline. The 50% shade treatment manifested a comparatively higher value of *P*n in contrast to the other treatment groups. Conversely, when the shade intensity reached or exceeded 80%, the value of *P*n was markedly lower than that of the non-shaded group (**Figure 3A**). The effects of varying shade intensities on stomatal conductance (gs) were also scrutinized. A propensity for an increase trailed by a decrease in gs was discerned with the augmentation of shade intensity, albeit the disparity was not statistically significant. The gs of the groups with shade intensities of 30% and 50% was marginally higher than that of the other groups, while the data for the remaining treatments were analogous to those of the non-shaded group (**Figure 3B**). The impact of shade on light use efficiency (LUE) and water use efficiency (WUE) was conspicuous and exhibited a parallel trend. LUE and WUE were both enhanced in all shaded groups in comparison to the control group, with LUE registering a more pronounced augmentation when the shade surpassed 50%. The 90% shade group exhibited a significantly superior performance level in contrast to the low-shade groups. No significant discrepancies were detected in water utilization among the shaded groups. However, a pronounced disparity was evident between the 70% shade group and the control group (**Figures 3C, F**).The impact of the diverse shade treatments on transpiration rate (*T*r) and photosynthetically active radiation (PAR) manifested comparable patterns, as attested by the observation that the values of the shaded groups were inferior to those of the control group and exhibited a diminishing trend with the intensification of shade intensity (**Figures 3D, E**).

### 3.3 Photosynthetic characteristics and OJIP curves of *P. polyphylla* under different moisture conditions

**Figure 4:**
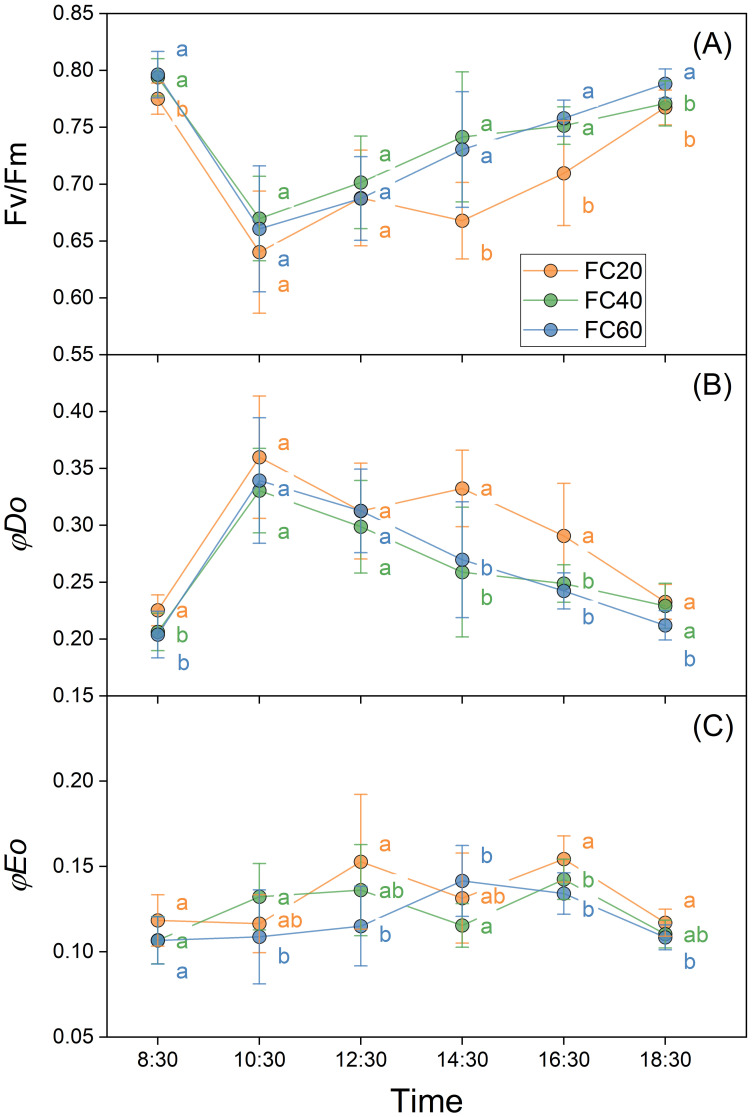
Daily variation of *Paris polyphylla* quantum yield of PSII (Fv/Fm, **A**), quantum yield for electron transport (φ*D*o, **B**) and quantum ratio foe heat dissipation (φ*E*o, **C**) in different field water capacity treatments. Values are means ± SE (n=6). FC20 to FC60 indicated the soil water content from 30% to 60%, respectively. Different letters indicate significant differences (*P*<0.05) in one-way ANOVA (Duncan test).

The trends in the alterations of PSII quantum yield (Fv/Fm) in reaction to diverse field water-holding capacities manifested a remarkably conspicuous similarity, presenting a pattern of an initial downtrend succeeded by a rebound (**Figure 4**). The peak value was discerned in the treatment groups at 10:30 a.m. Subsequently, a substantial disparity became evident between the FC20 group and the other groups. This was concurrent with a secondary diminution in Fv/Fm values at that juncture, trailed by yet another recovery (**Figure 4A**). The electron transport quantum yield (*φDo*) exhibited an opposing trend in comparison to that of Fv/Fm, demonstrating an initial upward tendency followed by a downward shift, with a zenith attained at 10:30 a.m. in all treatment groups. Nevertheless, the value of *φDo* in the FC20 group experienced a resurgence at 14:30 p.m., promptly trailed by an immediate decline (**Figure 4B**). The heat dissipation sub-ratios (*φEo*) of the assorted water control treatment groups displayed an upward and downward oscillation in the data over the course of time. To sum it up, no pronounced difference was detected in the *φEo* of the FC20 group at any temporal point. The FC40 group exhibited a minimal degree of fluctuation in *φEo* at all time instants, whereas the FC60 group evinced a significant deviation from the initial values at each time point (**Figure 4C**).

**Figure 5:**
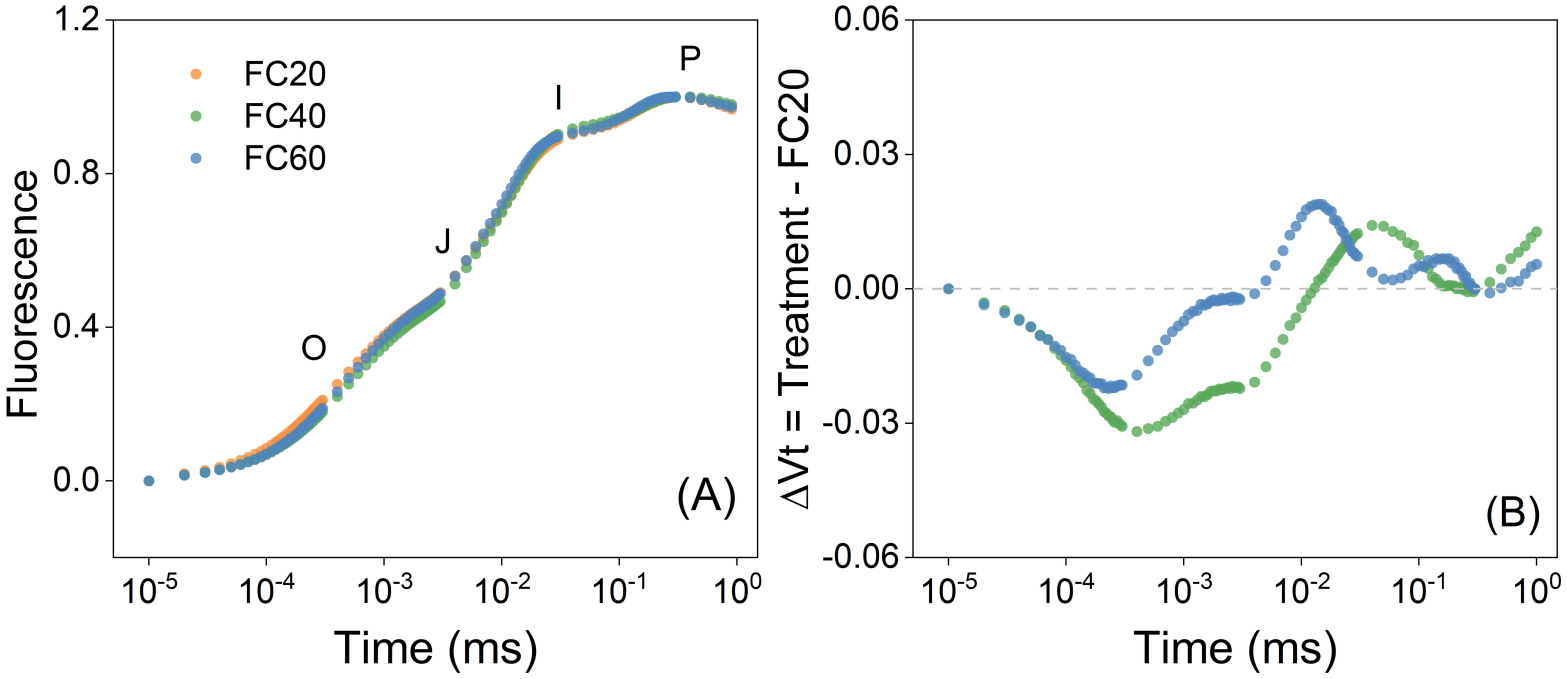
Chlorophyll a fluorescence induction curve of *Paris polyphylla* in different during development of photosynthetic apparatus **(A)** and the effect of development of PSII on the relative variable fluorescence **(B)** in different field water capacity treatments at 14:00 p.m. Vt = (Ft – Fo)/(Fm – Fo), ΔVt was calculated by comparing of different shading treatment and ambient treatment. Values are means. FC20 to FC60 indicated the soil field water capacity from 30% to 60%, respectively.

When the field water holding capacity ranged from 20% to 60%, the trends of the plant OJIP curves manifested a remarkable degree of resemblance (**Figure 5A**). This implies that the fluorescence-inducing capabilities of *P. polyphylla* leaves were relatively equivalent, and moreover, the photosynthetic apparatuses of the damaged leaves exhibited a comparable level of photosynthetic capacity. In contrast to the FC20 group, the relative variable fluorescence of the water-control FC60 group surpassed that of the water-control FC40 group (**Figure 5B**). A diminution in water content led to the inhibition of electron transfer.

**Figure 6:**
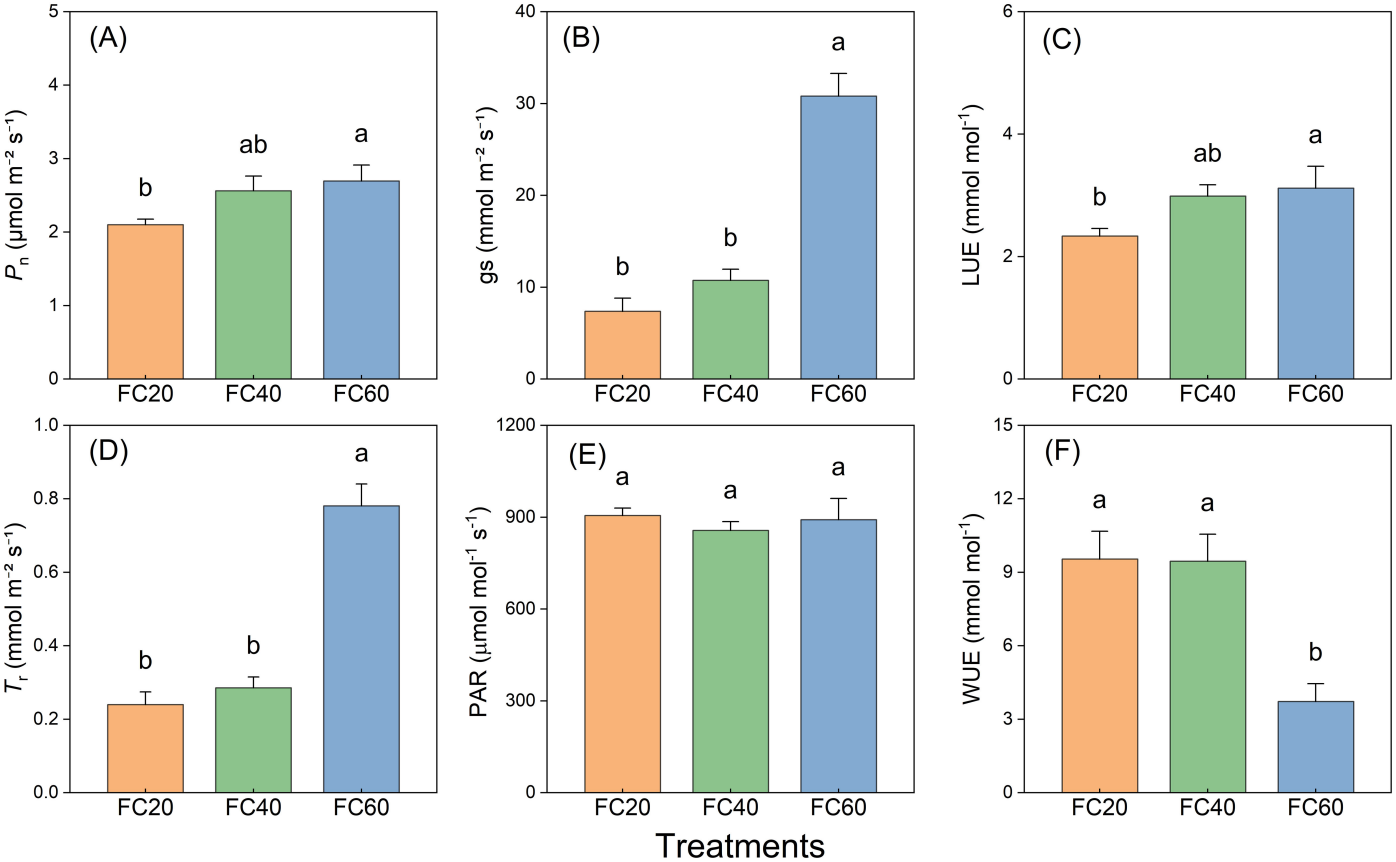
Field water capacity effects on *Paris polyphylla* net photosynthetic rate (*P*n, **A**), stomatal conducatance (gs, **B**), light use efficiency (LUE, **C**), transpiration rate (*Tr*, **D**), photosynthetically active radiation (PAR, **E**) and water use efficiency (WUE, **F**) in 10:00 a.m. Values are means ± SE (n=6). Horizontal coordinate indicate different soil moisture treatments, FC20 to FC60 indicate the soil field water capacity from 30% to 60%, respectively. Different letters indicate significant differences (*P*<0.05) in one-way ANOVA (Duncan test).

The diverse field water holding capacities exerted a perceptible influence on the net photosynthetic rate (*P*n), stomatal conductance (gs), light use efficiency (LUE), and transpiration rate (*T*r) of *P. polyphylla.* Comparable trends were discernible across all these variables (**Figure 6**). These impacts were most conspicuous in the FC60 group, trailed by the FC40 and FC20 groups in descending order of magnitude. The effect of soil moisture on gs and *T*r was especially remarkable, with the FC60 group manifesting the most substantial modifications. The experimental outcomes illustrated that the FC60 group registered significantly higher values in comparison to the other groups (**Figures 6A–D**). Additionally, the influence of the fluctuating field water holding capacity on photosynthetically active radiation (PAR) was not substantial, and the water control groups exhibited a similar effect (**Figure 6E**). Nevertheless, marked disparities were detected in water use efficiency (WUE) among different field water holding capacities, with the FC60 group presenting a considerably lower value than the other groups (**Figure 6F**).

### 3.4 Light response curve of *P. polyphylla* under different moisture conditions

**Figure 7:**
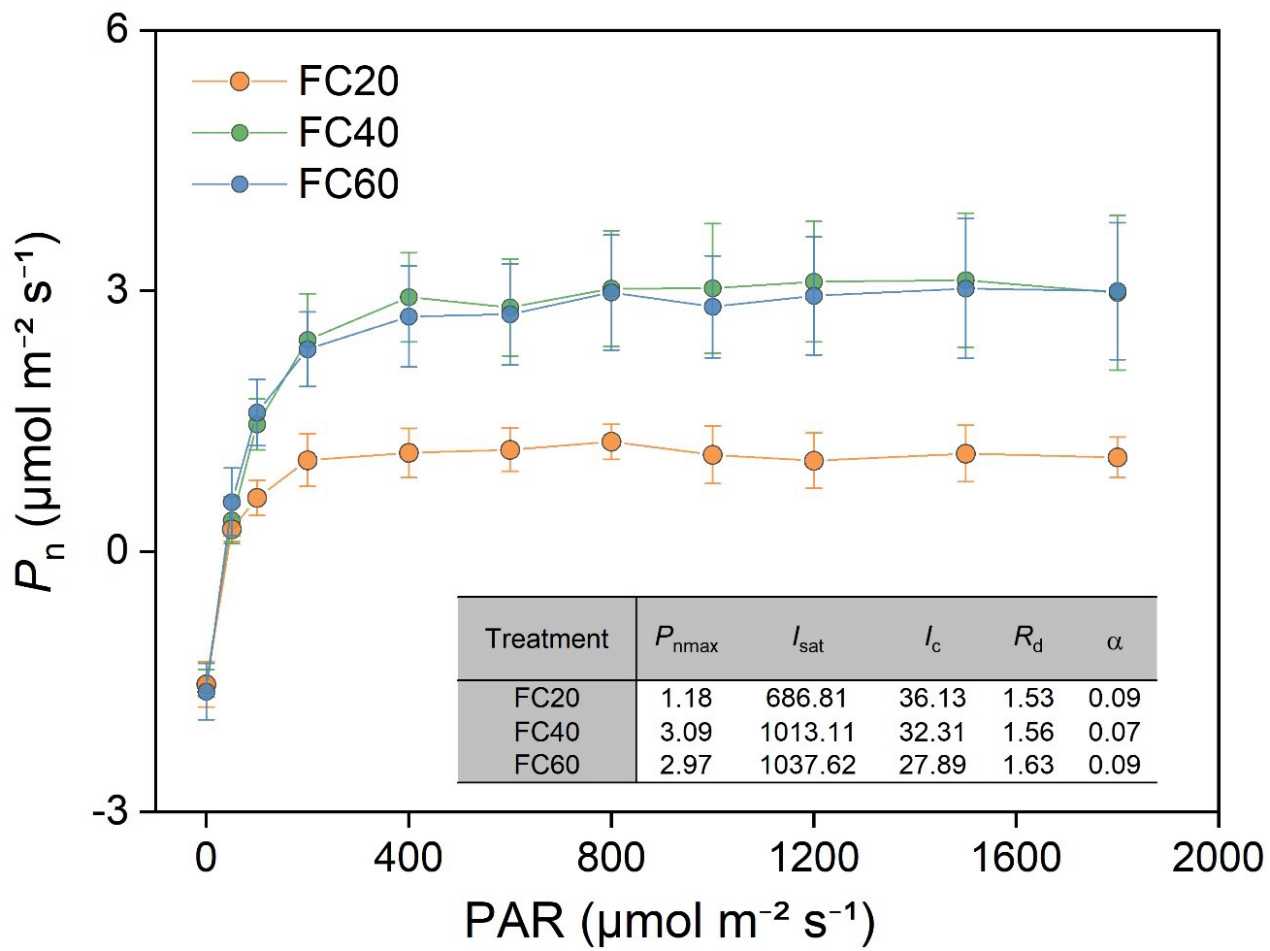
Relationship between photosynthetic rate (*P*_n_) and photosynthetically active radiation (PAR) of *Paris polyphylla* in different field water capacity treatments. Values are means ± SE (n=3). FC20 to FC60 indicated the soil field water capacity from 30% to 60%, respectively. *P*_nmax_, maximum net photosynthetic rate; *I*_sat_, satuation light intensity; *I*_c_, light compensation point; R_d_, dark respiration rate; α, apparent quantum yield of photosynthesis.

The light response curve of *P. polyphylla* manifested that soil moisture is capable of exerting an influence on the photosynthetic characteristics (**Figure 7**). Moreover, a low soil water content (FC20) could give rise to a significant divergence in the characteristic parameters of the light response curve. This result implies that the FC20 group exhibited a relatively diminished photosynthetic capacity in comparison with the other two groups. Additionally, the photosynthetic indices in the leaves of *P. polyphylla* presented conspicuous differences in response to the variations in field water holding capacity. Within the control scope of this experiment, an augmentation in field water holding capacity was concomitant with an elevation in the maximum net photosynthetic rate (*P*nmax), saturated light intensity (*I*sat), and dark respiration rate (*R*d) of the leaves. Conversely, the light compensation point (*I*c) demonstrated a decline.

## 4 Discussion

### 4.1 Shade levels influence on photosynthesis characteristics of *P. polyphylla*

Light serves as a crucial ecological factor, significantly influencing the morphology of plant leaves, their anatomical structure, and photosynthetic physiological characteristics (Zhang et al., 2022; Xue et al., 2023). It provides essential energy that enables plants to perform various physiological functions, including transpiration (*T*r) and photosynthesis (*P*n). *P*n facilitates nutrient accumulation and distribution throughout the plant, supporting growth and development, while *T*r generates a pulling force that transports water and minerals, supplying water for photosynthesis. This process also promotes water evaporation, which helps cool the plant, preventing heat damage and removing excess carbon dioxide to enhance photosynthesis (Haworth et al., 2018; Qu et al., 2024a). The ratio of variable fluorescence to maximum fluorescence (Fv/Fm) serves as an indicator of the photochemical efficiency of photosystem II (PSII); a decline in its value indicates inhibited electron transfer on the donor side of PSII. Research indicates that this fluorescence parameter remains stable under non-stress conditions, regardless of species or growth conditions, but decreases significantly under photoinhibition (Wang et al., 2022). Fv/Fm reflects the maximum photosynthetic capacity of plants, typically ranging from 0.79 to 0.84 under healthy conditions (De Micco et al., 2023). However, when light intensity surpasses a certain threshold, Fv/Fm decreases inversely with increasing light intensity. In this study, varying light intensities among treatment groups resulted in significant differences in Fv/Fm values for *P. polyphylla*. The marked decrease in Fv/Fm during daily photosynthetic dynamics, coupled with the inability of partial shading to restore initial values, suggests that the light intensity experienced under this shading was excessive, causing irreversible damage to the plants. Furthermore, the rapid increase in light intensity during midday resulted in photodamage to *P. polyphylla* (**Figure 1**), necessitating shade treatment for effective protection. The critical damage threshold was approximately 50% shade, equivalent to 816 μmol·m^-2^ ·s^-1^. Significant differences in Fv/Fm were observed between the unshaded and 30% shaded groups, indicating that light intensities above 600 μmol·m^-2^ ·s^-1^ adversely affected the plants, which were particularly sensitive to this range. Light intensity negatively correlates with light energy utilization and positively correlates with *T*r, further influencing gs (Johnson et al., 2015). As light intensity and temperature increase, photosynthetic enzyme activity and transpiration are affected, leading to water loss in chloroplasts, stomatal closure, and the “midday depression” phenomenon (Koyama and Takemoto, 2014). High light intensity reduces stomatal conductance; however, to mitigate heat damage, plants increase transpiration rates, resulting in excessive water loss and a decrease in photosynthetic raw materials, thereby inhibiting photosynthesis (Zhong et al., 2023). Conversely, low light intensity leads to reduced stomatal conductance and insufficient transpiration, limiting the water supply for photosynthesis. Optimal light intensity ensures maximum gs, moderate *T*r, and high LUE and WUE, thereby maximizing *P*n (Pignon et al., 2021).

*P. polyphylla* is a shade-tolerant plant, and light significantly influences photosynthetic indicators. *P*n and gs are typically correlated, although this correlation is nonlinear (Hussain et al., 2024b). As gs increases, the rate of increase in *P*n gradually diminishes (Drake et al., 2017; Niu et al., 2023; Wang et al., 2023). Furthermore, stronger photosynthetically active radiation can affect photosynthetic efficiency (Gitelson et al., 2021) by regulating the degree of stomatal opening and closing (Huang et al., 2024). During the initial stage of plant sensitization, which serves as a preparatory phase, stomata are critical for physiological activities related to water allocation (Marchin et al., 2023), enabling photosynthesis and transpiration to function normally and achieving an initial equilibrium in homeostasis (Upadhyay et al., 2013). As light intensity increases, gs rises, leading to enhancements in *T*r and *P*n until gs reaches its maximum. When light intensity exceeds a certain threshold, increased temperature results in greater *T*r (Sadok et al., 2021), which reduces the water vapor pressure deficit in the leaves (Yuan et al., 2019). Consequently, stomata close, and gs decreases rapidly, disrupting the equilibrium. This disruption results in reduced gas exchange and water circulation, causing an excessive rise in transpiration rate and depletion of water resources (Sadok et al., 2021). Simultaneously, due to stomatal limitations, CO_2_ influx decreases, becoming insufficient to sustain normal photosynthesis, which leads to a reduced photosynthetic rate. In summary, *P. polyphylla* is a shade-loving plant that prefers warm conditions and does not require high light levels. It thrives in indirect or diffuse light while avoiding direct sunlight (Wang et al., 2011). Shade plants exhibit enhanced light capture and utilization efficiency, but excessive light intensity can inhibit growth (Goh et al., 2012). Rapid fluctuations in light intensity significantly affect *P. polyphylla*, leading to PSII photoinhibition and a reduction in light energy capture, absorption, and conversion (Wu et al., 2021). In natural environments, short bursts of high light stress at noon are typically survivable due to the plants’ recovery abilities and various photoprotective mechanisms (Shi et al., 2022). However, prolonged exposure to high light intensity can cause irreversible damage. Prior studies suggest that *P. polyphylla* efficiently utilizes low light, with artificial cultivation benefiting from 70%-90% shade to optimize photosynthetic potential (Hong et al., 2020). While *P. polyphylla* excels in low light conditions, excessively low light can hinder nutrient accumulation. Our experiment determined that not all indices improve with increased shade intensity. As shade increased, light and effective radiation decreased, but LUE increased, peaking at 50% shade, which yielded the highest net photosynthetic rate with minimal intergroup differences. In conclusion, for artificial cultivation, 50% shade is optimal, followed by 30% and 70%, as excessive light intensity causes photoinhibition, negatively impacting Fv/Fm values and resulting in varying degrees of light damage.

### 4.2 Soil moisture influences on photosynthesis characteristics of *P. polyphylla*

In this study, *P*n of three groups of plants exhibited similarities across various soil moisture environments (**Figure 6**). However, under short-term water control, a water content of 20% (FC20) induced mild drought stress in *P. polyphylla*. Under such stress, the plants demonstrated lower photosynthetic indices while maintaining Fv/Fm (**Figure 5**), indicating a healthy physiological state. Consequently, a soil water content of 20% may represent the lower limit of water requirements for *P. polyphylla*. As soil water content increased, the net photosynthetic activity of *P. polyphylla* improved, suggesting a positive correlation between higher moisture levels and the net photosynthetic rate. The absence of decline inflection points within the water content range of 20% to 60% implies that the upper water limit for *P. polyphylla* is at least 60%. Therefore, the optimal water threshold for *P. polyphylla* is estimated to be between 20% and 60% soil water content, indicating a relatively broad ecological amplitude and classifying it as a hydrophilic plant. Both the OJIP curve and the 820 nm light absorption technique effectively compare the primary photochemical reaction activity and energy transfer efficiency of different plant leaves under non-injurious conditions (Luo et al., 2022). Research has demonstrated that Fv/Fm remains relatively stable under normal physiological conditions, albeit subject to subtle variations in response to environmental changes (Ibaraki and Murakami, 2007; Jägerbrand and Kudo, 2016). The similarity of OJIP induction curves among plants under varying moisture conditions in this study suggests that the photosynthetic capacity of each group remains intact, with approximately equivalent maximum photosynthetic capacities. Reduced soil moisture content correlated with decreased Fv/Fm (Takashima et al., 2021) and electron transfer quantum yield (φDo), while increasing the heat dissipation quantum ratio (φEo) in *P. polyphylla*. Conversely, higher soil moisture levels enhanced the efficiency of light energy captured by PSII for photochemical reactions, thereby influencing energy distribution within photosystem II (Hamerlynck and O’Connor, 2021). Under low humidity conditions, electron transfer from QA to QB on the PSII electron acceptor side is significantly inhibited, and lower water content increases the likelihood of PSII electron transfer blockage in plants (Sharma et al., 2020). Moderate to severe drought stress further impedes electron transfer and diminishes leaf activity, thereby impairing the plant’s capacity to release oxygen through photosynthesis, as well as reducing chloroplast reactions and chlorophyll fluorescence intensity (Sharma et al., 2020). Moderate to severe drought stress further impedes electron transfer and diminished leaf activity, thereby impairing the plant’s capacity to release oxygen through photosynthesis, as well as reducing chloroplast reactions and chlorophyll fluorescence intensity (Skotnica et al., 2000; Zhuang et al., 2020), ultimately damaging the physiological functions of photosynthetic organs (Fu, 2018). The initial fluorescence parameter (Fo) and Fv/Fm of the light reaction center in *P. polyphylla* significantly decreased with declining moisture content (Tribulato et al., 2019), indicating a high sensitivity of the light reaction center to moisture conditions. Increased moisture enhances the activity of the light reaction center (Sharma et al., 2020). Thus, from a physiological perspective, water availability critically affects the electron transfer processes in plant photosynthesis; drought stress impedes these processes, damaging the physiological functions of photosynthetic organs and adversely impacting overall plant photosynthesis. Conversely, improved water conditions can mitigate damage and facilitate recovery to a higher physiological activity state.

Transpiration generates a pull that facilitates the transport of water and minerals, while also providing the necessary water for photosynthesis. Additionally, the evaporation of water aids in temperature regulation, preventing heat stress and excessive accumulation of carbon dioxide, which can inhibit respiration and photosynthesis (Zhang et al., 2018; Qu et al., 2024b). This study demonstrates that decreased water availability in *P. polyphylla* adversely affects both *P*n and *T*r, with more pronounced effects observed at lower water content. Conversely, higher water availability correlates with increased net photosynthetic and transpiration rates, thereby enhancing plant stability. Furthermore, comparisons of light energy utilization among different *P. polyphylla* specimens indicate that higher water content improves light energy utilization rates. The light response curve reveals that increased water content lowers the light compensation point while raising the light saturation point, reflecting enhanced photosynthetic activity and metabolic rates (**Figure 7**). Environments with medium to high humidity exhibit broader light utilization capabilities in *P. polyphylla*, enabling plants to effectively absorb and utilize light energy (Xia et al., 2017). WUE serves as a critical physiological index, with higher WUE indicating more efficient water use. In this experiment, higher water content corresponded to increased transpiration rates but lower WUE. Consequently, while *P. polyphylla* exhibited higher transpiration rates under high humidity conditions, its water utilization rate was lower; in contrast, both transpiration and water utilization rates were more balanced under medium humidity conditions.

## 5 Conclusion and prospect

The findings of this study indicate that a shade intensity of 50% is optimal for the growth of *P. polyphylla*, as evidenced by the analysis of its photosynthetic parameters under the specified experimental conditions. Additionally, a shade intensity of 70% was also identified as favorable. Under medium humidity and moisture conditions, *P. polyphylla* exhibited the most advantageous photosynthetic characteristics. Furthermore, a moisture content ranging from 40% to 60% was found to be most conducive to the normal growth and development of *P. polyphylla*. Consequently, during the actual planting process, it is essential to monitor environmental factors in real-time to identify discrepancies between these factors and critical values. This monitoring facilitates the implementation of appropriate shade interventions, as well as irrigation or drainage when ambient light intensity and soil moisture reach specific thresholds, thereby mitigating potential damage to the plants. Moreover, our results suggest that appropriate management becomes increasingly necessary as the *P. polyphylla* plant industry faces challenges associated with climate change, including high temperatures, elevated light intensity, and drought, which are likely to become more prevalent in the future. However, it is important to acknowledge that in this study, light intensity and soil water content were primarily designed as independent factors, lacking consideration of their interaction. Ecological factors are mutually influential and intertwined, indicating that the effects of light and moisture interact in a complex and integrated manner. Further research necessitates an analytical approach that comprehensively accounts for multiple ecological variables. Additionally, beyond light and soil moisture, other ecological factors affecting the photosynthetic characteristics and physiological functions of plants must be considered, such as soil temperature, microbial environments, organic matter content, and pH values (Michaela et al., 2022; Ogée, 2024; Verma et al., 2024). There remains a vast area of unexplored territory concerning the implications of ecological factors on the photosynthetic attributes of *P. polyphylla*. The optimal shading level and soil water content are likely to differ by location; thus, it is crucial to foster a deeper understanding of the complex coupling effects and to employ methods that are aligned with local circumstances.

## Data Availability

The original contributions presented in the study are included in the article/**Supplementary Material**. Further inquiries can be directed to the corresponding authors.
